# Book Reviews

**Published:** 1824-08

**Authors:** 


### MEDICAL AND SCIENTIFIC BIBLIOGRAPHY.

Vexat censura . . . . . . Corvos.

Art. I.—*Pathological Observations on the Rotated and Contorted Spine, commonly called Lateral Curvature, deduced from Practice, —in which are shown the Causes that produce it; the Reason for its being mistaken for an Incurvation of the Spinal Column; and the Means best adapted to its Prevention and Cure, agreeably to the Principles laid down, and the Author's Experience.* By Andrew Dods, M.D. late of Edinburgh, and Surgeon in the Royal Navy.—pp. 239. Cadell, London; Blackwood, Edinburgh. 1824.

The author of this work has some very peculiar opinions on the causes, appearances, and modes of treating, lateral distortions of the spine. He seems to consider this kind of deformity as arising, not from girls sitting carelessly or awry, but from their sitting in an erect posture. This, however, is not so extraordinary a deviation from the opinions commonly entertained as that which he expresses regarding the condition of a girl who would by most persons be regarded as crooked. Our author informs us, that she is not really crooked, merely labouring under a changed aspect of the natural profile, or semi-profile, of the spine; that, in short, surgeons have all along been deceived, and that the curve is but " visual, as well as a manual, deception." The mode of treatment is consistent with the views which the author entertains of the causes of distortion; for it differs from that of every other surgeon, in so far that a concave surface is recommended instead of a plane, when it is intended to give rest and ease to the muscles of the spine.

We shall lay before our readers some extracts from the work, to show the views which the author has formed, and the manner in which he treats the subject.

After some remarks on the importance of the subject, and the injury arising from the restraints imposed on their persons by devotees at the shrine of fashion, the author proceeds—

"Whoever, then, shall impose a restraint upon it (the body) in this respect, must be considered guilty of a breach of the laws of nature, and shall not fail to be punished for his temerity. Whoever shall, either accidentally or wilfully, deprive any of his members of motion, or, what is the same thing, who shall fail to exercise the functions of its muscles, will assuredly distort such member, and lose the use of it." (p. 52.)

We cannot afford space for the long discussion on scrofula, nor for the reasons which have induced the author to call the malady "contracted spine;" but we shall follow him into his description of the state of the spine when it has become crooked, and here we shall find that he has some ideas peculiar to himself.

"This," says the author, at page 155, "is the difficulty which has hitherto presented itself, but which I have now surmounted, and hope on philosophical as well as anatomical principle; shewing what have been all along considered curvatures of the spinal column is but a changed aspect of its natural flexures, which are brought into profile, or rather semi-profile, by the aggregate rotatory movement of the vertebræ upon each other; the consequence of the permanent contraction of the muscles that move them, produced by malposition: and that, therefore, medical men have been striving all along to account for what does not exist, the whole being a visual, as well as a manual, deception."

The author admits that the view which he offers of spinal distortions, differs from what has been generally given; and upon this he very honestly, and with some simplicity, makes the following observations:

"As some corroborating proof will naturally be looked for, I shall, as the best mode of affording it, candidly describe the manner in which I was first led to a knowledge of it myself, with my reasoning thereon, and what I resorted to, to prove it.

"Having been induced, of late years, to apply myself more particularly to the investigation of this interesting deformity, and being previously convinced, in my own mind, that it depended more upon muscular contraction, brought on by the fashionable modes and customs of the day, than upon structural disease of the vertebræ, I was led to the adoption of friction for its cure; and, in order that I might give it the fairest trial possible, I was induced to become the operator myself.

"During the course of my operations upon several patients, I was struck in all of them (for they were all contorted to the right side,) with a considerable bony hardness and projection on the left side of the loins, raised nearly to a level with the spinous processes; and this I found to be the case in the patients whose spine exhibited little or no apparent curvature, as well as in those in whom the apparent curvature was very great. Being led to investigate this anomaly, I redoubled my exertions to discover the cause; and I found, after the muscles had been relaxed by friction, (for they were in every case extremely rigid,) that the bony projection was the transverse processes of the vertebræ of the loins, which I could now as distinctly feel and count as the spinous. From this circumstance I was led to examine whether I could feel the transverse processes of the same vertebræ on the opposite side; but without effect, for they appeared to have sunk inwards, completely out of reach."

Dr. Dods seems to have puzzled himself unnecessarily about "organic" and "functional" contraction of muscles. From the following passage our readers may form some idea of his difficulties:

"Any one who will take the trouble to examine into the state of the muscles of the back, while the body is stretched upon either of these planes, the horizontal or inclined, must plainly perceive, I think, that they approach nearly to the greatest degree of their organic contractility; and that it is quite impossible for them to be in the slightest manner relaxed, so long as the person continues in their extended position." (p. 145.)

This most extraordinary doctrine, that a person is not at rest while lying flat on the back, will be found to have been the idea constantly operating on Dr. Dod's mind, during the whole of his inquiry. At page

166, he states that, " as the production of spinal distortion has been accounted for purely on the principle of muscular contraction, so must the cure be conducted solely on that of muscular relaxation;" and, at page 213, we have a repetition of this sentiment, and, at the same time, the announcement of the principle which he follows in the treatment.

" I hold it to be an axiom in physic, as well as surgery, that to cure a disease is to remove the cause. As spinal contortion, then, depends on the continued extension of the body, and the contraction of the spinal muscles thereby produced, so, therefore, this being the cause, it must be removed before we can possibly remove the contortion. Now, there is no way, with which I am acquainted, of doing this so effectually, as by bending forward the body and relaxing the spinal muscles; so, therefore, after frictions, I place my patients on their backs upon a concave elastic couch."

It would be easy to show how completely the author is mistaken in supposing that the muscles of the spine are relaxed, when a patient is laid in this position; but it is unnecessary to enter into any discussion on a question, which can be decided at once by any of our readers, who choose to make the experiment of lying for ten minutes on such a couch as the author recommends.

---

ART. II.—*An Enquiry into the Causes of the Curvatures of the Spine; with Suggestions as to the Means of preventing, or, when formed, of removing, the Lateral Curvature.* By T. JARROLD, M.D.—pp. 147; two Plates. Longman and Co. London.

ON our first looking through the pages of this work, we were a little astonished at the style in which several of the cases were given; but, on referring to the Preface, we found an explanation,—viz. that it had been adapted for the general reader. The same circumstance will, perhaps, explain how it comes to contain opinions so entirely at variance with those generally received.

The volume contains some remarks on the natural structure of the spine,—on carious spine,—on the hip-joint disease,—and on anomalous diseases of the spine; but, as the author announces his work as a treatise on *Lateral Curvature*, we shall confine ourselves to the task of giving our readers some idea of the notions which he entertains on this, at present, very popular question. In doing this we shall avail ourselves of the author's own words, and the following quotations will show that he considers lateral curvature to depend not only on *disease*, but on a specific, and even an hereditary, malady.

" In the view which I take of the subject, a specific disease distends the cartilages, which the energies of the constitution frequently removes, and the figure suffers only in degree. In other and more advanced cases, the distention is so considerable as to occasion an uneasy sensation about the part, the effort to relieve which causes the curvature. It is not that the ligaments, or the bones, or the muscles, are diseased, or that the disease of the cartilages is of itself the cause; but, the muscles being made to act partially on the spine while the cartilages are in this state, the same effect follows as when pressure is applied obliquely to the ankle." (P. 88.)

" In a review of the origin and progress of this malady, connected with the appearance after death, it is evident that disease, not debility, originates the lateral curvature." (p. 89.)

"Were it not a specific disease, it might, like the outward curvature, be accidental and solitary; one individual of a family might be its victim: but it spreads its influence through the blood, and, like the gout, though it slumbers, it has not ceased. Every stage, from the elevated shoulder to positive crookedness, may be found in one generation; in the next it may not appear, but returns in the third." (P. 95.)

Although these doctrines were not calculated to raise our opinion of the author's pathological acumen, we confess we were not prepared for the flight of imagination which presents itself when we come to the treatment. It appears that Dr. Jarrold regards curvature of the spine as bearing a strict analogy to bronchocele, and even to be capable of cure by similar means.

"The diseases of the cartilages and of the bronchial glands most *affect* females: both commence in youth, and are not excited or retarded by other diseases. This seeming relation led to the use of the same means of cure, and with the most marked and beneficial result, so as to justify the expectation that, at no distant period, the lateral curvature will, like the scars of the small-pox, be a reproach to the parents, rather than a necessary evil." (P. 94.)

"It has been my object to prove that the lateral curvature has its origin in a specific constitutional disease, and consequently requires a constitutional remedy. And, as I have before hinted that there might be some relation between it and bronchocele, I have made use of similar remedies: from ten to fifteen grains of burnt sponge, and from four to six grains of carbonate of soda; and, if debility be considerable, twenty drops of nitric acid are directed to be given daily. Very soon the increased flesh on the shoulders begins to diminish, and in two or three weeks disappears. The shoulder-blades at the same time fall, and re-occupy their natural situations. The health, which had been more or less disturbed, resumes its ordinary state; the mind becomes cheerful, and capable of application; the languid, dispirited aspect, which seemed to call for the use of tonics and stimulants, is dispelled without them. Medical treatment is seldom further required, unless the appetite and digestion be impaired." (P. 117.)

We have in this Number presented to our readers two works on the Diseases of the Spine, which certainly have the merit of containing some original ideas; and we repeat that we have communicated them almost entirely in the author's own words. In one, the author insists that the distorted spine is but a visual as well as a manual deception, and that surgeons have all along been mistaken in supposing that, because a person is crooked, the spine is curved. In the other, the author informs the world that the hump on the shoulder originates in causes similar to those of bronchocele; and, consequently, may be removed (sometimes in three weeks) by burnt sponge and soda!

---

ART. III.—*An Elementary System of Physiology.* By JOHN BOSTOCK, M.D. F.R.S. &c. Vol. I.—8vo. pp. 518. London: Baldwin, Cradock, and Joy. 1824.

WE are aware that Dr. BOSTOCK has been some time employed in collecting materials for this work, from among the extensive resources which this author commands. His avowed object is to present the physiological student with a concise view of the present state of the science, without endeavouring to compile a popular treatise; and yet, at the same time, it is intended for the use of the general reader. The first volume has issued from the press alone, and contains a portion only of the whole work.

A work of this description has long been esteemed as a great desideratum, and must prove eminently useful in our days, when new experiments, new views and facts, are continually pouring in upon us from many quarters, and are sufficiently numerous and various to hold the practised student in doubt, and utterly to confound the ex-professionally learned.

It is evident that the accomplishment of such a task requires no small degree of industry and judgment, when widely-scattered facts have to be collected together, and the dross to be carefully sifted from the ore. The gleanings of the physiological labours of the past and present age, certainly afford a rich and abundant harvest, perhaps little less important than that which has, of late years, sprung more suddenly from the researches of modern chemistry. How far those changes which result from recent inquiries affect the practical application of medicine generally, it is difficult to determine; and how much improvement physiology has experienced, we must leave posterity to settle for themselves. The present century finds the science freed from fetters which bound it in the last; but it may be said, that it has since run a wild unchecked career, and which it will probably continue to pursue, till sober reason and experience can limit the boundaries of discovery.

Dr. Bostock, in the first place, endeavours to clear the ground before him, by referring to the doctrines of the ancients, and pointing the reader's attention to the successive theories and systems formed at different periods to the present age. To effect this, he selects the most popular authors of each succeeding era, affording us concise reviews of the opinions and discoveries of Hippocrates, Aristotle, Galen, Stahl, Hoffmann, Boerhaave, Haller, Cullen, Hunter, and Bichat; noticing how one doctrine gave place to another, and the changes produced in the science by every new view, as it arose.

The plan of the work is next developed. He divides all the functions of the living body into two grand divisions: the one relating to the *contractile*, and the other to the *sensitive* properties; the first belonging to *muscular*, and the second to *nervous* matter. The first division embraces the individual functions dependent upon *contraction*, the circulation of the blood, respiration, animal temperature, secretion, digestion, assimilation, absorption, and generation; all of which are preceded by an account of the nature of the two powers of contractility and sensibility, and of the organs by which they are exercised,—the muscles and the nerves: and, as bone and membrane constitute the basis of the body, a description of these is premised.

The contractile functions, as above enumerated, being described, it is next intended to review the subjects of the second great division, the *sensitive* functions. This division includes what are termed the five senses,—sight, hearing, smell, taste, and touch. There will also be added to these some other classes of sensation, equally specific in their nature, but less understood and recognized. The most important of these latter are the sensation which accompanies muscular contraction, that of heat and cold, and that of hunger.

Another class of phenomena, of a compound nature, in which a sensitive function is succeeded by some intellectual operation, or in which an intellectual operation produces a change in the corporeal organs, will next be noticed. This class, however, the author intends to treat briefly, for fear of encroaching upon the province of the metaphysician,—one which, he wisely foresees, would lead him far astray from the boundaries of physiology. Accordingly, he will limit himself to those phenomena only indirectly connected with the science of physiology,—such as the effects of association, habit, imagination, sympathy, and volition. But the author does not stop here; he is tempted to handle the delicate subject of craniology,—and we wish him well through it. Some observations upon the much-controverted point of the nature of the connexion between the intellectual faculties and the organ by which they are exercised, are connected with craniology; and he concludes with some remarks upon the natural progress of the animal body, from the commencement of its existence, through its state of maturity, to its decline and final dissolution.

The present volume goes no further than the circulation of the blood: our criticism must, therefore, be deferred chiefly till the completion of the whole work.

As to the arrangement adopted, every author seems to have his own fancy upon this point; and, although there is scarcely any system quite free from objections, yet it is, perhaps, a matter of less importance than usually supposed. We should ourselves have given precedence to the nervous function, because all others are either directly or indirectly governed by it. Whilst, therefore, we admit that all vital actions are referable either to contractility or sensibility, we object to all the functions being embraced by two grand divisions, separating these two general functions as the immediate causes of the others, but independent of each other; for we see not how contraction, whether voluntary or automatic, can be considered independent altogether of nervous influence: a proposition inferred, if not intended to be so, to all appearance, by the arrangement adopted.

The author treats his subject in a manner highly useful to students, by referring to authorities, so as to form one condensed history of physiology, and points out, with a masterly hand, where credit and reliance may be given, and where it should be withheld. This plan naturally gives his style an air of pedantry, and it may, perhaps, appear complicated and tedious to many. As a work of reference, however, it is highly valuable, and ought to have a place on the shelves of every medical library. It pretends to no display of descriptive language; it is, therefore, written with more laboured caution than eloquence. We cannot help thinking that a clearer arrangement might be adopted for students; and many points referred to in the progress of the work, might have been more conveniently considered in one general introductory view, prefixed to the history of physiology, with which the work is introduced. For example, the student's mind might have been made clear upon the connexion between life and organization, and the definitions and examples of these might have preceded, instead of being subsequently referred to, when occasion required; as also how far the exact sciences are applicable to the living body; and the degrees of obedience which the vital powers pay to physical laws, with their mutual influence over each other, when combined together.

From the multitude of opinions cited on most points, an objection, very common to compilations, exists, from the great liability of the inexperienced being perplexed, and made to waver in their belief. Certainly, the subjects of this volume are of a most controverted nature. We question whether the London pupil will find time to imbibe all the instruction which this work affords; and either, therefore, a less extensively learned work, more upon the scale of a course of lectures, would not be preferable as a companion to the studies of anatomy, surgery, and medicine. The present publication may do better when, in after-life, the pupil has leisure to con it over by his library fireside. Certainly the deepest physiologist will find it useful to peruse Dr. Bostock's "Elements of Physiology," as it combines a vastly extended body of facts and theories, with a copious reference to authorities.

We quote the following, as a specimen of the author's mode of treating a controverted point, in reference to the sensibility of membrane:—

"With respect to the sensibility of membrane, considered as a matter of fact, independent of any speculation concerning the nature of organization or vitality, the opinions of physiologists have been various; and it was especially the subject of a warm controversy, about the middle of the last century, between Haller and Whytt. As is often the case in points of this nature, the question has been decided by a kind of compromise: it is generally admitted, with Haller, that simple membrane is insensible in its healthy and natural state, but that it is liable to inflammation, and that it then becomes sometimes exquisitely painful. The cause of this fact, the excessive degree of pains which are excited by disease in parts that are at other times without sensation, is, perhaps, not altogether understood. We may remark concerning it, that one effect of inflammation is to enlarge the bulk of the inflamed part, and the pain is generally in proportion to the difficulty with which the part admits of this extension. A high degree of inflammation may exist in loose cellular texture, and we may be scarcely sensible of its existence; while the inflammation of the periosteum of the smallest bone, as of a tooth, of the sclerotic coat of the eye, or of the tense membrane about the finger-nail, will be almost intolerable. In these cases, we shall probably always find that, even if the inflamed part be without nervous filaments, which give it sensibility, still there are some branches of nerves immediately contiguous to it, which, in consequence of the firmness of some of the neighbouring parts, are pressed upon and irritated, while the blood-vessels connected with them are in a state of plethora; for it seems to be a general law of the animal economy, that no cause is more powerful in producing pain than a certain degree of pressure upon a nerve, while its sensibility is augmented by an unusual determination of blood.

"It is probable that much error and confusion took place on the subject of the sensibility of membrane, among the anatomists and physiologists, after the revival of letters, in consequence of their blind veneration for the ancients. Hippocrates, who had but an imperfect knowledge of the existence and use of nerves, confounded them (or at least placed them in the same class) with the tendons, from some similarity in their visible structure and appearance; and, having observed very serious effects to ensue from injuries of the proper nerves, he laid it down as a maxim, that tendons and other membranous parts are among the most sensible organs of the body. This erroneous opinion materially influenced, not only physiological speculations, but medical and surgical practice, even as late as the middle of the last century, long after the distinction between nerves and tendons was thoroughly understood. Even Boerhaave fully subscribed to this doctrine, in which he was warmly seconded by his learned, but obsequious commentator, Van Swieten; and the influence of the old hypothesis upon our language may still be observed in the present day.

"John Bell, in his usual animated and impressive manner, describes the dreadful effects which this opinion concerning the great sensibility of membrane, formerly produced in the operation of lithotomy. As the bladder principally consists of membrane, it was agreed by all the learned operators, for a succession of ages, that it would be improper to cut or divide any part of it; and, therefore, in order to extract the calculus, a variety of instruments were employed for the purpose, as it was said, of dilatation, but which, in fact, caused the most cruel laceration of the organ itself, and of the neighbouring parts. It is truly astonishing to observe how the weight of authority bore down the clearest dictates of reason, and the most decisive results of experience; and how the most obvious facts were warped and misconstrued, before mankind would submit to prefer the evidence of their own senses to the mere hypothetical opinions of the ancients.

"From these remarks it will appear that, except bone, membrane may be regarded as the most simple in its properties of all the organized parts of the body. By this expression, I must be understood to mean that the properties which belong to it are likewise found in many other natural objects. Cohesion necessarily belongs to all solids, while flexibility, extensibility, and elasticity, are possessed by many vegetable and some mineral substances, and also by dead animal matter; whereas, spontaneous contractility and sensibility are the exclusive properties of the living body. We are, however, as much unacquainted with the intimate nature and immediate cause of the properties of membrane as of contractility and sensibility; only we are much more familiar with these operations." (P. 36—39.)

At present, the most interesting points of physiology seem to be the discoveries of the separate functions of the nerves in their distribution, as pointed out by Bell, &c.; and in their radical origins from the spinal cord, as proved by Magendie; and also the highly important discoveries, published by Flourens, relative to the seats of sensation and irritation in the brain, a subject upon which Rolando, Haller, &c. experimented, but not with the success which has recently attended the experiments of the French physiologists. Upon these points, we quote the following remarks of the author:—

"Although we have so little certain knowledge respecting the exact parts of the brain, which may serve as the respective centres of the two functions of perception and volition, some very interesting experiments have been lately made by Mr. Bell and M. Magendie, which confirm the opinion that had been advanced by physiologists as a plausible conjecture, that the transmission of these two powers from the extremities to the brain, or from the brain to the extremities, is effected by different nerves, or at least by different nervous filaments. The nerves that proceed from the spine have a double origin, or are composed of two nerves, —one proceeding from the anterior, and the other from the posterior, part of the cord. Now, it has been found, by direct experiment, that the parts to which the nerves are sent are deprived of motion and sensation respectively, according as the anterior or posterior roots of the nerves are divided. From this separation of the functions of the nerves, and the appropriation of each function to a separate organ, we gain an analogical argument for the same kind of separation in the brain; and this conjecture would appear to be sanctioned by the experiments of M. Flourens and M. Rolando.

"A very interesting series of facts has been lately brought forward by Mr. Bell, respecting the functions of the nerves, which throws considerable light upon their mode of action, and their connexion with the other parts of the system. There are certain circumstances in the anatomical structure and the distribution of the nerves, which led to the arrangement of them into two classes, and which indicated that they each serve different purposes in the animal economy. From the situation of these two sets of nerves, with respect to their origin and to each other, Mr. Bell has given them the names of symmetrical or original, and irregular or superadded. The first set (which might perhaps be called, more appropriately, the general nerves,) consist of the fifth pair of the cranial, and all the spinal nerves; they have double roots, one of which is connected with ganglia; they pass laterally to the two halves of the body, the two sides having no connexion with each other, and they are distributed to all the muscular parts that are under the control of the will. They appear to be the organs of perception and volition, deriving, as we may conjecture, these two functions from their double roots. These we may regard as that part of the nervous system which serves the purpose of establishing our connexion with the external world.

"Mr. Bell's second set of nerves proceed by single roots from the base of the medulla oblongata, or the parts immediately connected with it; they proceed in a much more irregular manner than the former, and are distributed to all the organs which are concerned, either directly or indirectly, in the function of respiration. From this circumstance they have received the denomination of respiratory nerves, as well as that of superadded or irregular. Their course is designated by this last term, as they pass from one organ to another in the most intricate manner,—connecting them together, passing across the general nerves, occasionally uniting with them, and forming the connecting link between the two halves of the body. These nerves are not under the control of the will, and are not capable of exciting perception; they are, therefore, furnished only with the faculty of transmitting the nervous influence, or with what Dr. Philip styles nervous power, in opposition to sensorial. Some very curious and important pathological deductions have been made by Mr. Bell, from the new views which he has given us on the subject of the nerves; and we have also a number of additional remarks by Mr. Shaw, which confirm and illustrate Mr. Bell's doctrines, and give us reason to expect that they may be applied with great advantage to the practice of medicine and surgery.

"The result of our observations upon the nervous system and its functions is, that it has two distinct powers, that of receiving and transmitting impressions, which is exercised by the nerves and spinal cord, and that of perception and volition, which is more immediately exercised by the brain. Upon this principle Blumenbach has arranged the organs of these functions into the two classes of sensorial, comprehending the brain and its immediate appendages, and the nervous, properly so called, including the nerves, the plexuses, and the ganglia. The sensorial organs are the exclusive seat of the powers of perception and volition, and of the intellectual faculties; while the office of the nerves is to serve as media of communication between the common centre and the organs of sense and motion." (P. 280—284.)

In conclusion we beg to observe, that, although we cannot hazard a determinate opinion upon the merits and utility of a work so unfinished as this at present is, yet, if the subsequent parts are equal to that published, we have no hesitation in declaring it to be our opinion, that it is the best elementary work of the kind in existence, and one of the greatest utility to the profession at large, as a work of deep and accurate research and extensive reference.

Not a word of our Physiology

Art. IV.—*The Animal Kingdom, arranged in conformity with its Organization.* By the Baron Cuvier, &c. &c. &c. *With additional Descriptions of the Species hitherto named, of many not before noticed, and other original Matter.* By Edward Griffith, F.L.S. and others.—8vo. pp. 203; with eighteen Plates. London: G. and W. B. Whittaker.

Notwithstanding that we find "Natural History" enumerated in our title-page among the objects of this Journal, yet such is the press of matter more purely professional, that we are seldom able to turn from the beaten path of medicine into those more varied fields of investigation, into which we would naturally be led by works similar to that now under our consideration. Zoology, indeed, is a study which seems scarcely to have flourished among us: at least, its cultivation has not hitherto given rise to any works of pre-eminent merit; and we imagine that, while in the other branches of science we can bring their equals to the best in Europe, yet, in this department, the names of Buffon, Cuvier, St. Hillaire, and Rudolphi, are unrivalled by any we can oppose to them. Indeed, almost the only systematic work on Zoology we possess is that of Dr. Shaw, which cannot be regarded as complete, or commensurate with recent discoveries. We have, therefore, much satisfaction in making known the objects of the present work, as far as our circulation extends, giving to it whatever benefit can result from the humble meed of our commendation.

The title-page expresses pretty clearly the design of the learned translator, which is, in fact, to give the whole of Cuvier's "Regne Animal," with such additions as may render it interesting as a general zoological biography. The intimate connexion of such pursuits with the legitimate objects of our Journal, is almost too obvious to require illustration; but the words of Cuvier, in his Preface, are so much to the point, that we cannot resist our inclination to quote them. "I was necessitated," says he, "in furtherance of my object, to make anatomy and zoology, dissection and classification, proceed hand in hand together; in my first remarks on organization, to look for the best general principles of distribution; to employ those principles in making new observations, and those new observations, in their turn, to carry to perfection the general principles of distribution. In fine, to produce from this action and re-action of the two sciences such a system of zoology as might serve for an introduction and a guide in anatomical researches, and such a body of anatomy as might tend to develop and explain the zoological system."

Particular Physics, or Natural History, – Of Living Beings, and of Organization in general,—Division of organized Beings into Animals and Vegetables,—Of the Forms peculiar to the organic Elements of the Animal Body, and of the principal Combinations of Chemical Elements,—Of the active Forces of the Animal Body,—A Summary Consideration of the Functions and Organs of the Animal Body, and of the various Degrees of their Composition,—A rapid Sketch of the Intellectual Functions of Animals,—Of Method or System, in its application to the Animal Kingdom,—General Distribution of the Animal Kingdom into four grand Divisions.

This last section we shall quote, both as giving a concise view of the great divisions adopted by Cuvier in his "Regne Animal," and at the same time illustrating the manner in which the translation is executed.

"*General Distribution of the Animal Kingdom into four grand Divisions.*

"If we divest ourselves of prejudices founded on the divisions of the animal kingdom formerly recognised, and consider animals without reference to their relative size or utility, our own degree of knowledge respecting them, or any other extraneous circumstances, we shall find that there are four principal forms, after which all living beings appear to have been modelled. The basis of these distinctions is laid on the nature and organization of the several creatures themselves: the ulterior divisions of them, with whatever names they may have been decorated, are but slight modifications of the primary; and consist entirely in the addition or development of certain parts, which make no essential change in the general character of their conformation.

"In the first of these general forms or models, including that proper to man, and the animals resembling him most nearly, the brain and the chief trunk of the nervous system are enclosed in bony coverings,—the former called the cranium, and the latter the vertebra. To the sides of the vertebra, as to a central column, are attached the ribs and the bones of those limbs which form, as it were, the framework or carpentry of the body. The muscles, generally speaking, form a second covering for the bones which they put into action; and the viscera are enclosed in the head and trunk.

"Creatures of this form are denominated 'vertebrated animals,' (*animalia vertebrata.*)

"These have all red blood, a muscular heart, a mouth with two horizontal jaws, distinct organs of vision, smell, hearing, and of taste, situated in cavities of the head, and never more than four limbs. The sexes in these animals are invariably separated, and a similar distribution prevails among them of the medullary masses, and of the principal branches of the nervous system.

"On a close examination of each of the parts of this grand system, we shall discover a general analogy of conformation, even in the species most remote from each other; and can easily trace the gradations of the same plan from man to the lowest of the fish.

"In the conformation peculiar to the second grand division of living beings, we find no skeleton. The muscles are simply attached to the skin, which forms a soft and contractile covering, from which proceeds, in several of the species, a scaly or laminous substance, called shells, the position and production of which are analogous to those of the mucous body. Within this general envelope are the viscera and nervous system, which last is composed of many scattered masses, attached together by nervous threads. The chief of these masses, placed in the œsophagus, receives the denomination of the brain. Of the senses, properly so called, we can seldom distinguish, among these animals, more than the organs for those of taste and vision; and we sometimes find that even these are wanting. One family alone exhibits the organs of hearing. In other respects, this division is characterised by a complete circulating system, and peculiar organs of respiration. The apparatus for digestion and secretion are scarcely less complicated than are those of the vertebrata.

"We give to the animals, whose conformation is modelled according to this second form, the appellation of Mollusca.

"Although the general plan of their organization is not so uniform as that of the vertebrata in relation to external configuration of parts, yet even here the degree of resemblance is generally analogous, both as to structure and functions.

"The third general form is that of insects, worms, &c. Their nervous system consists of two cords extending along the belly, and swelled out, at regular intervals, into knots or ganglia. The first of these, placed on the œsophagus, though called the brain, is not much larger than the rest. The covering of their body is divided by transverse folds into a certain number of rings, the teguments of which are in some hard, and in others soft; but the muscles are invariably attached to their interior. We often find articulated limbs attached to the sides of the body or trunk, but it is as frequently destitute of any.

"This division we denominate 'articulated animals,' (*animalia articulata.*)

"It is in these animals that we can observe the transition from the circulating system in closed vessels, to a nutritive process performed by simple imbibition; and likewise a transition corresponding to this from the respiratory system in organs confined to certain parts, to the same operation performed through the medium of trachiæ, or air-vessels dispersed through the entire body. The organs of taste and sight are the most distinct among the articulated animals. A single tribe possesses those of hearing. The jaws of this division, when any are to be found, are invariably lateral.

"The fourth and last form comprehends the entire of those animals usually known under the name of zoophytes, and which may also be termed, with propriety, 'radiated animals,' (*animalia radiata.*)

"In the three divisions preceding this, the organs of motion and sensation are symmetrically disposed, as it were, on the two respective sides of a certain axis. In this last, similar organs have a circular arrangement round a common centre. The zoophytes, in truth, approach nearly to the homogeneous character of plants. They possess neither a nervous system sufficiently distinct, nor particular organs of sensation. In a few of them we may discover, with difficulty, some vestiges of circulation. Their respiratory organs are generally upon the surface of the body. The intestines of the great majority consist of a sort of bag, through which there is no passage; and those which are lowest in the animated series exhibit nothing but a kind of homogeneous pulp, possessed of motion and sensibility."—(P. 60—64.)

We next have the first grand division of the animal kingdom, viz. the *vertebrated animals;* first in the list of which stand the *Mammalia.* Prevented, as we are, by our limits, from being minute, we shall content ourselves with selecting a few facts with regard to the anatomy of this class, which are common to them all. The upper jaw is fixed to the cranium, the lower being articulated to a condyle; the neck (with one exception) is composed of seven vertebræ; the anterior ribs are attached to a sternum; the anterior extremity to a shoulder-blade, which is not articulated with any other bone, though frequently resting on the sternum through the medium of a clavicle. The posterior extremity is articulated with a pelvis.

The brain is composed of two hemispheres; and contains a corpus callosum, two lateral ventricles, corpora striata, thalami optici, and tubercula quadrigemina. Between the optic thalamii is a third ventricle, communicating with a fourth beneath the cerebellum. They have all the prominence denominated *tuber annulare.*

The eye is always lodged in a socket, and has two lids, with more or less of a third. The ear is always provided with a tympanum and membrana tympani; four small bones, a vestibule, semicircular canals, and cochlea. The cranium is divided into three compartments. The tongue is always fleshy, and is attached to a hyoid bone. There is a velum palati, which establishes a direct communication between the larynx and back part of the nostrils. The organ of voice in all is situated in the upper part of the trachea. The lungs are two in number, and are enclosed, without adhering, between the ribs and diaphragm. The intestinal canal is suspended by one fold of the peritoneum, while another hangs in front of them.

The generation of the mammalia is viviparous, and the young are nourished for some time after their birth with milk; a secretion peculiar to the class.

Next in order comes the division of the class Mammalia into orders: first follows

The *Bimana*, or man, the only genus in the order to which he belongs, and zoologically distinguished by having hands at the upper extremities only; the posterior being employed in walking.

The *Quadrumana*, having hands at the four extremities.

*Carnivora*, so called from the nature of their food; they have no thumb capable of free motion, or of being opposed to the rest.—These three orders have three different kinds of teeth,—viz. molar, canine, and incisor.

*Glires*, having extremities similar to the preceding, but wanting the canine teeth, and having incisors in the front of the mouth adapted for a peculiar kind of mastication.

*Edentata*, so called from the defects of their teeth; the incisors being absent, some wanting the canine, and some having no teeth at all. Their extremities are much cramped, and they have large claws.

*Ruminantia*, known by the cloven foot, the want of incisors in the upper jaw, and the presence of four stomachs: as the name implies, they ruminate.

*Pachydermata* includes the rest of the hoofed mammalia, and are distinguished by the thickness of their hide.

*Cetacea*. This constitutes the last order of the mammalia: they have no posterior extremities; they live in the water, and are remarkable for their gigantic size.

In conclusion, we have a short sketch of the natural history of man, by Cuvier, as the first genus of his first class and order; and a supplemental history of the same animal, by the translator. These investigations have been thought to lead to opinions hostile to the received doctrines of religion; and on this point Cuvier has behaved rather jesuitically in all his writings, not caring to commit himself one way or other. His translator, however, has determined not to incur any suspicion, being an open opponent to those who have enlisted science under the banner of scepticism.

We have thus given an outline of the contents of this first portion of Mr. Griffith's undertaking, and we have done so, not without the hope of inducing some of our readers to seek for further information in the "Animal Kingdom," convinced as we are of the direct tendency of such studies to open to us a vast field of discovery in the physiology of the human frame.

